# Coaxial Electrospinning Construction Si@C Core–Shell Nanofibers for Advanced Flexible Lithium-Ion Batteries

**DOI:** 10.3390/nano11123454

**Published:** 2021-12-20

**Authors:** Li Zeng, Hongxue Xi, Xingang Liu, Chuhong Zhang

**Affiliations:** State Key Laboratory of Polymer Materials Engineering, Polymer Research Institute, Sichuan University, Chengdu 610065, China; zengli_0718@foxmail.com (L.Z.); hongxue@scu.edu.cn (H.X.)

**Keywords:** lithium-ion battery, silicon anode, electrospinning, core-shell

## Abstract

Silicon (Si) is expected to be a high-energy anode for the next generation of lithium-ion batteries (LIBs). However, the large volume change along with the severe capacity degradation during the cycling process is still a barrier for its practical application. Herein, we successfully construct flexible silicon/carbon nanofibers with a core–shell structure via a facile coaxial electrospinning technique. The resultant Si@C nanofibers (Si@C NFs) are composed of a hard carbon shell and the Si-embedded amorphous carbon core framework demonstrates an initial reversible capacity of 1162.8 mAh g^−1^ at 0.1 A g^−1^ with a retained capacity of 762.0 mAh g^−1^ after 100 cycles. In addition, flexible LIBs assembled with Si@C NFs were hardly impacted under an extreme bending state, illustrating excellent electrochemical performance. The impressive performances are attributed to the high electric conductivity and structural stability of the porous carbon fibers with a hierarchical porous structure, indicating that the novel Si@C NFs fabricated using this electrospinning technique have great potential for advanced flexible energy storage.

## 1. Introduction

Lithium-ion batteries (LIBs), as one of the most promising energy storage systems, are being used in many applications, from portable electronic devices to electric vehicles and other power storage platforms [1,2,3]. However, the commercial graphite anode with its limited theoretical capacity (372 mAhg^−1^) deadlocks the long-term development of LIBs [4]. Among all other anode materials, silicon is garnering a great deal of attention due to its high theoretical capacity (about 4200 mAh g^−1^) and its low working potential (~0.5 V vs. Li/Li^+^) [5]. Nevertheless, commercial applications of Si-based anodes are hampered due to their poor cyclic ability, caused by the huge volume variation (>300%) and aggregation during the alloying process, leading to pulverization of the anode as well as the severe detachment of active materials [6,7]. As one of the semiconductor materials, Si shows low electrical conductivity, resulting in poor rate capability [8]. In order to resolve the problems mentioned above, a valid strategy is to design a hybrid electrode. Conductive materials, such as graphene [9,10], carbon nanotubes [11,12], and Mxene [13], have been widely researched to improve the electrochemical properties of Si due to their excellent conductivity and good mechanical properties.

On the other hand, motivated by the rapid development of wearable portable electronics, high energy density storage devices with excellent flexibility have attracted growing attention [14,15]. However, conventional LIBs are fabricated via a slurry-casting method [16], and the necessary current collector and binder significantly increases the total weight of the battery, meanwhile, the active materials are much easier to detach from the current collector under extreme deformation [17,18]. Among all the composite structures, the concept of constructing one-dimensional nanofibrils has been accepted due to their good electrochemical and mechanical flexibility [19,20,21], making them attractive as alternative free-standing electrodes.

Recently, electrospinning has emerged as a facile and effective method for fabricating freestanding one-dimensional nanofibers and is widely used for anode materials of batteries [22,23,24], where active materials are dispersed into a polymer precursor solution to produce composite electrodes. Hans et al. [25] reported novel amorphous silicon/carbon (Si/C)-based particles produced using an industrially scalable gas phase synthesis method and thermal decomposition; the Si/C composite delivered an initial capacity of 3070 mAh g^−1^. Although the electrochemical performance of the obtained anode was greatly improved, the uneven distribution of Si NPs and the random coverage of the carbon matrix also resulted in a poor cyclic stability [26]. Therefore, there is still a need to explore the available methods of preparing a stable Si anode with a good rate performance.

In this work, a core–shell structure was designed to improve the electrochemical performance of a Si-based electrode using a facile co-axial electrospinning technique. The novel and hierarchical structure of the obtained core–shell Si-C nanofibrils (Si@C NFs) combined a compact carbon shell and a porous carbon core embedded with Si NPs, making it possible to achieve a high specific capacity, superior cycling stability and good rate capability. As expected, this designed Si@C NF composite electrode exhibits a high capacity of 762.0 mAh g^−1^ at 0.1 A g^−1^ after 100 cycles. 

## 2. Materials and Experiments 

Si nanoparticles (NPs) (diameter: 60–100 nm), polyvinylpyrrolidone (PVP), poly (methyl methacrylate) (PMMA), and N-dimethylformamide (DMF) were all purchased from Sigma Aldrich Inc., Shanghai, China.

### 2.1. Preparation of Si@C NF Electrode

The precursor core–shell Si/PMMA-PVP nanofibers were obtained via a coaxial electrospinning technique with 15 wt.% Si NPs added to an inner dispersion of 8 wt.% PMMA and DMF solvent. The outer shell solution was a uniform mixture of 8 wt.% PVP and DMF solvent. Both the core and shell solutions were stirred at 60 °C for 24 h, followed by ultrasonic treatment for 1 h. Typically, the as-prepared homogeneous electrospinning suspensions were loaded into a 10-mL syringe in the electrospinning device (Beijing Yongkang Co., Beijing, China). The flow rates of the inner dispersion and outer solution were 0.3 and 0.6 mL h^−1^, respectively, the voltage was controlled at 14 kV, and the distance of the needle to the rotary aluminum foil collector was 15 cm. For comparison, Si/PVP composite nanofibers were also prepared using the uniaxial electrospinning method with the 15 wt.% Si dispersed under a flow rate of 0.6 mL h^−1^ and a voltage of 14 kV. The prepared Si/PVP and core–shell Si/PMMA-PVP composite nanofibers were stabilized in air at 150 °C for 24 h, further stabilized at 250 °C for 12 h under a heating rate of 2 °C min^−1^, and then carbonized at 800 °C for 2 h to finally form the Si/C NF and Si@C NF electrodes. 

### 2.2. Materials Characterizations

The morphology of the prepared nanofibers was observed by high-resolution transmission electron microscopy with an accelerating voltage of 200 kV and a resolution of 0.1–0.2 nm (HRTEM, Tecnai, FEI company, Hillsboro, OR, USA) and scanning electron microscopy with an accelerating voltage of 30 kV and a resolution of 1.0 nm (FESEM, Quanta, FEI company, Hillsboro, OR, USA). The crystal structures of the samples were characterized by X-ray diffraction (XRD) analysis on a Rigaku Smart Lab diffractometer with a scan range from 10° to 80°. The surface area was measured using the Brunauer–Emmett–Teller (BET) method on a Micromeritics Tristar 3020 in a N_2_ atmosphere. Thermogravimetric analysis was conducted on a TGA-Q50 (TA Instrument Co., Ltd., Selb, Karlsruhe, Germany) under an air atmosphere with a heating rate of 10 °C min^−1^ raised from room temperature to 700 °C.

### 2.3. Electrochemical Tests 

The electrochemical properties of Si/C NF and Si@C NF electrodes were measured in CR2032 coin cells (Shenzhen Kejing Equipment Co., Ltd., Shenzhen, Guangdong, China). The composite electrode film was cut into pieces which directly used the anode without additional binders or conductive additives. The coin-type cells were assembled under a high pure argon atmosphere in a glove box (H_2_O < 0.5 ppm, O_2_ < 0.5 ppm) using Li foil as the counter electrode, Celgard 2325 porous film as the separator and 1.0 M LiPF_6_ dissolved in a mixture of ethylene carbonate and diethyl carbonate (EC/DEC, 1:1, *v*/*v*) as the electrolyte. Subsequently, the galvanostatic charge/discharge properties of the cells were recorded using an automatic battery test system (Land CT2001A, Wuhan Shenglan Electronic Technology Co., Ltd., Wuhan, China) at various current densities in the voltage range of 0.01–1.5 V. Cyclic voltammetry (CV) was tested in the potential range of 0.01–1.5 V (vs. Li/Li^+^) at a scan rate of 0.1 mV s^−1^; electrochemical impedance spectroscopy (EIS) measurements were performed using a Biologic VMP3 electrochemical workstation at a frequency range of 10^−2^–10^5^ Hz with an amplitude of 5 mV. Finally, a flexible battery was assembled with a free-standing Si@C NF anode, Li metal film counter electrode, current collectors, LiPF_6_-based electrolyte, and flexible plastic shell.

## 3. Results and Discussion

**Figure 1:**
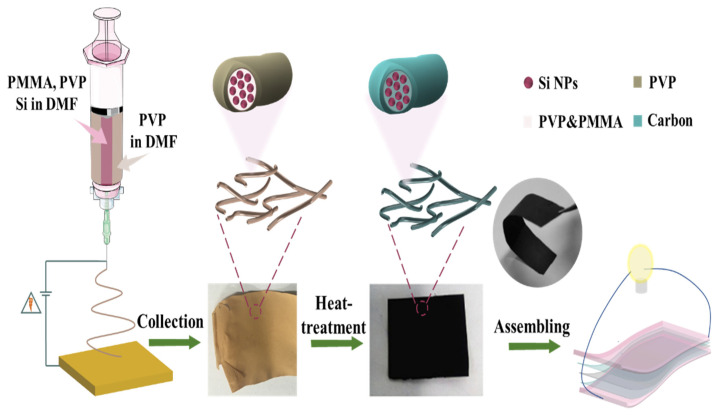
Schematic illustration of preparation for flexible Si@C NFs using coaxial electrospinning technology.

The synthesis process of the Si@C NF electrode with specific core–shell structure is illustrated in Figure 1. Firstly, the precursor Si/PMMA-PVP was obtained by adding core silicon component (inner layer) and the shell non-silicon component (outer layer) into the two needles of different diameters and then solidified under the strong electrostatic field. As the generation of the core–shell structure was mainly due to the different pyrolysis behaviors of polymer, the following depolymerization of the core PMMA was kept at 150 °C while the shell PVP layer was still stable during the heat treatment. Continuous thermal-oxidation stabilization carried out at 250 °C could promote a series of chemical reactions, such as dehydrogenation and a cyclization process, which can stabilize the fiber structure. The final heat-treatment at 800 °C caused the polymer components to decompose thoroughly and finally form a hollow-shaped flexible Si@C NFs electrode.

**Figure 2:**
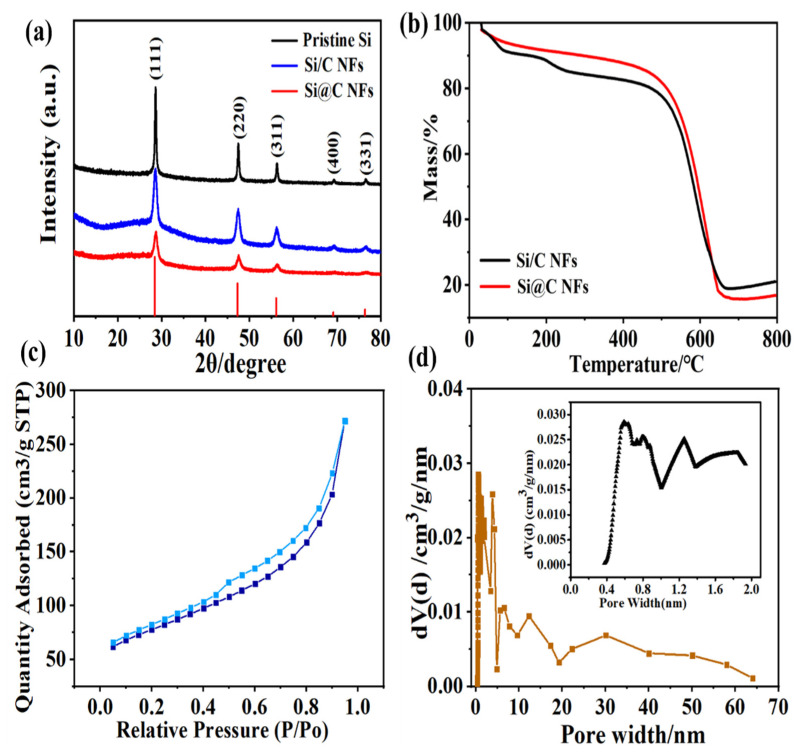
(**a**) XRD patterns of pristine Si NPs, Si@C NFs and Si/C NFs; (**b**) TG curves of Si@C NFs and Si/C NFs at a heating rate of 10 °C min^−1^ in N_2_. (**c**) The N_2_ adsorption/desorption isotherm and (**d**) pore size distribution of Si@C NFs.

The phase composition and crystal structure of the pristine Si NPs, the Si@C NFs, and the Si/C NFs were determined using XRD. As shown in Figure 2a, all samples exhibit obvious peaks at 2θ of 28.4°, 47.4°, 56.2°, 69.2°, and 76.5°, which are ascribed to the (1 1 1), (2 2 0), (3 1 1), (4 0 0), and (3 3 1) planes of Si (JCPDS: 27–1402). Note that there is no silicon oxide or Si-C alloy peak, indicating that Si was not oxidized or did not react with carbon during the continuous heating process. In order to acquire the precise percentage of Si in the Si@C NF and the Si/C NF samples, TGA analysis was performed in air in a temperature range from room temperature to 700 °C at a heating rate of 10 °C min^−1^. As shown in Figure 2b, an evident weight loss takes place in the range of 475–650 °C, which identifies the oxidation process of the pyrolytic carbon. The deduced mass percentages of Si in Si@C NFs and the Si/C NFs are about 16.8 wt.% and 17.8 wt.%, respectively. 

As the pore structure of the electrode has a great influence on its electrochemical performance, the pore structure of Si@C NFs was characterized using N_2_ isothermal adsorption/desorption measurements (Figure 2c). Calculated using the DFT method, the specific surface area of Si@C NFs is 120 m^2^g^−1^ with a total pore volume of 0.173 cm^3^g^−1^. The pore size distribution in Figure 2d indicates a typical hierarchical pore structure including micro-, meso- and macropores. The micropores mainly derived from the gas escape of the carbon matrix via the pyrolysis process of PVP component, while the larger meso- and macropores were mainly caused by the removing of PMMA and PAN component of the inner layer [27]. This hierarchical pore structure makes it easy for the permeation of electrolyte and the migration of Li ions, ensuring the better rate performance of electrode.

**Figure 3:**
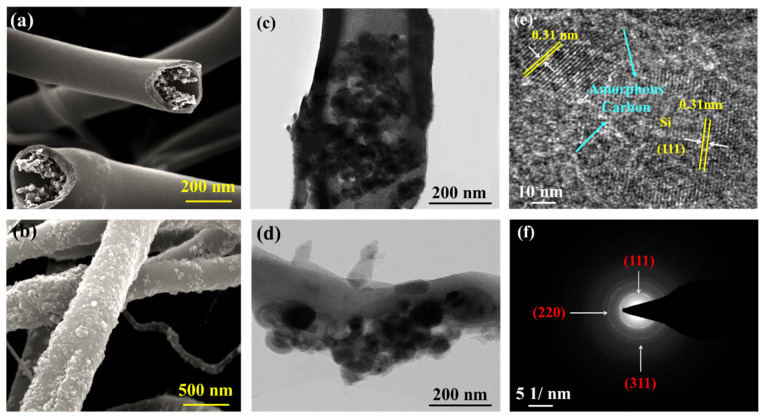
The SEM images of (**a**) Si@C NFs and (**b**) Si/C NFs; TEM images of (**c**) Si@C NFs and (**d**) Si/C NFs at low magnification; HMTEM image (**e**) and corresponding SAED (**f**) of Si@C NFs.

The corresponding morphologies of the Si@C NFs and Si/C NFs were investigated using SEM and TEM. Obviously, the vast majority of particles with a diameter of 200–500 nm were evenly dispersed in the core area of Si@C NFs (Figure 3a) and the Si@C NFs exhibited a much smoother surface with only very few Si NPs exposed outside, while amounts of Si NPs aggregated and were trapped on the outer surface of the Si/C NFs sample (Figure 3b and Figure S1). In order to further determine the distribution of Si NPs and the microstructure of the carbon, TEM analyses were performed on the Si@C NF and Si/C NF samples. For Si@C NFs, it can be observed from Figure 3b that the Si NPs are well encapsulated in the firm carbon shell with a thickness less than 100 nm, which can efficiently restrain the volume change. In contrast, the Si NPs of the Si/C NFs (Figure 3d) are scattered and completely clustered on the outside of the fiber. The composition of the inner layer of the Si@C NFs was further investigated using HRTEM. The 0.31-nm lattice fringe spacing in Figure 3e may be identical to the (111) plane of Si, and the SAED image (Figure 3f) shows a distinct diffraction ring of typical (111), (220), and (311) planes of Si, which is in accordance with the XRD result. It is worth noting that the Si NPs in Figure 3e are encased in the amorphous carbon, which builds bridges between the homo-disperse Si NPs and forms conductive pathways to ensure rapid electron transmission. Hence, the well-formed core–shell structure not only accommodates the volume expansion of Si NPs during the charge and discharge process, but also improve the conductivity of the composite electrode.

**Figure 4:**
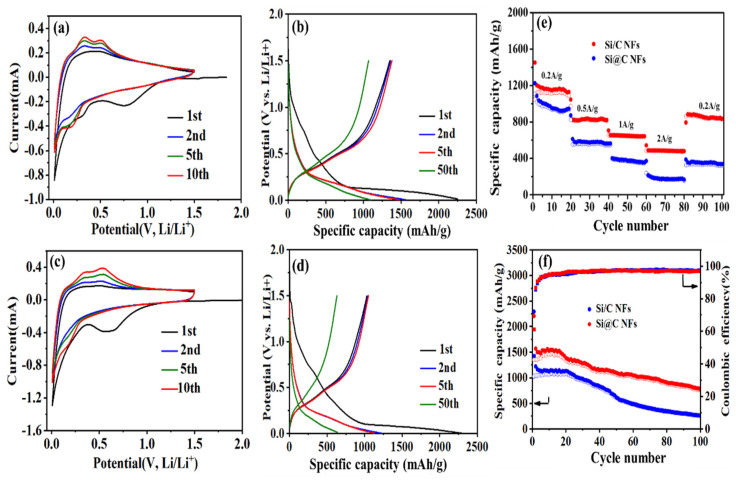
Cyclic voltammograms curves of (**a**) Si@C NF and (**c**) Si/C NF electrodes; galvanostatic charge/discharge profiles of (**b**) Si@C NF and (**d**) Si/C NF electrodes; (**e**) rate capabilities of Si@C NFs and Si/C NFs electrodes at a current density of 0.2–2A g^−1^; (**f**) cycling performances of Si@C NFs and Si/C NFs electrodes at a current density of 0.1 Ag^−1^.

The electrochemical performances of Si@C NF and Si/C NF electrodes are shown in Figure 4. The CV curves of Si@C NF and Si/C NF electrodes were tested at a scan rate of 0.1 mV s^−1^ with a potential range of 0.01–1.5 V (Figure 4a,c). As shown, during the cathodic process, the Si@C NFs electrode exhibited a small sharp cathodic peak at ~0.75 V, which disappears in following cycles, indicating an irreversible electrochemical reaction generated by the formation of a solid electrolyte interface (SEI) film [28]. This weak peak means that few side reactions were restrained by the surrounding amorphous carbon. Another sharp cathodic peak at ~0.2 V corresponds to the transformation from crystallite Si to amorphous LixSi alloy [29]. Obviously, the wide cathodic peak of Si@C NFs at 0.01–0.2 V is much weaker than that of the Si/C NFs, which means that the amorphous carbon suppresses the irreversible consumption of Li^+^. During the anodic sweep, two peaks at 0.38 and 0.55 V of the Si@C NF electrode correspond to the delithiation process. The intensities of all the peaks grow with the increased cycle number in the gradual activation process of electrodes. Without the protection of the solid shell, the pure Si electrode suffers rapid capacity decay in subsequent cycles due to the serious volume expansion occurring during the charging and discharging process (Figure S2a).

The discharge/charge voltage profiles of the Si@C NF and Si/C NF electrodes at a current density of 0.1 A g^−1^ are shown in Figure 4b,d. In the first cycle, a long discharge plateau at 0.75 V gradually disappears in subsequent cycles, attributed to the formation of an SEI layer, which is also consistent with the CV results. The flat plateau at 0.1 V is characteristic of lithiation of crystalline Si. Remarkably, in the first cycle, the Si@C NF anode delivers discharge and charge capacities of 2251.1 and 1378.6 mAh g^−1^ with an initial coulombic efficiency of 61.2%, which is much higher than that of the Si/C NFs (45.9%) and that of the pure Si electrode (54.5%, Figure S2b). During subsequent cycles, the charging and discharging curves of Si@C NFs electrode becomes smooth and no obvious voltage platform appears. The subsequent 50th cycle curve of Si@C NFs coincides well with the 2nd cycle curve with a discharge capacity of 1113.2 mAh g^−1^, indicating good cycle stability. However, both the Si/C NFs and pure Si electrodes suffer severe capacity loss from the detachment caused by the inevitable volume expansion during the charging and discharging process, and only retain a capacity of 628.4 mAh g^−1^ and 156.1 mAh g^−1^ after 50 cycles, respectively.

The rate capabilities of Si@C NFs and Si/C NFs electrodes were tested by the stepwise increasing current density from 200 mAg^−1^ to 2 A g^−1^. As demonstrated in Figure 4e, the Si@C NFs always exhibits stable capacity at different current density, the reversible capacities are 1162.8 mAh g^−1^, 805.6 mAh g^−1^, 623.9 mAh g^−1^, and 479.7 mAh g^−1^ at current density of 0.2, 0.5, 1, and 2 A g^−1^, respectively, and finally recover to 823.5 mAh g^−1^ when the current density reset at 0.2 A g^−1^. As a comparison, the Si/C NFs electrode delivers a poor rate performance with the reversible capacities of 981.2 mAh g^−1^, 568.1 mAh g^−1^, 382.6 mAh g^−1^, and 194.5 mAh g^−1^ at current density of 0.2, 0.5, 1, and 2 A g^−1^, respectively. In addition, the cycling performances of Si@C NFs and Si/C NFs at a current density of 0.1 A g^−1^ are also evaluated. As shown in Figure 4f, the Si/C NF electrode shows worse cycling stability than Si@C NFs and the capacity is decreased from 1051.3 to 261.8 mAh g^−1^ after 100 cycles; the successional capacity decay is due to the aggregation of Si particles on the surface of the carbon fiber matrix, which is completely expose to the electrolyte and is easily detach from the fiber without the protection of a solid carbon shell. On the other hand, the Si@C NFs electrode shows excellent cycling stability after 100 cycles, and the Si@C NF electrode maintains a reversible capacity of 762.0 mAh g^−1^. The superior stability of the Si@C NFs electrode is mainly ascribed to the novel core–shell structure, where the solid carbon shell avoids the direct contact of Si NPs and electrolyte, the porous amorphous carbon in the core buffer the volume expansion of Si NPs, and enhances the electronical conductivity of the composite electrode.

**Figure 5:**
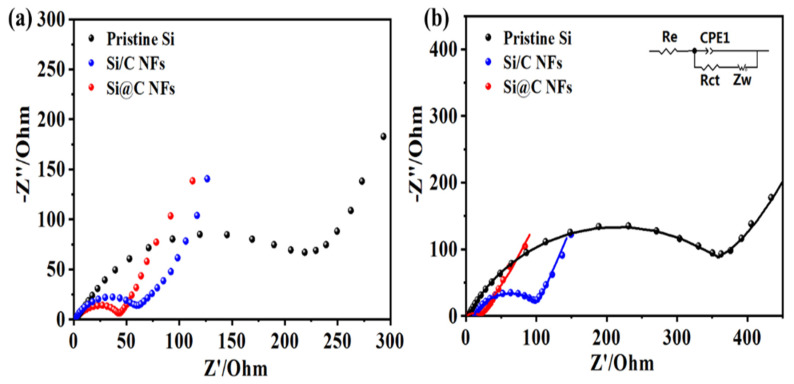
Nyquist plots for pristine Si NPs, Si/C NFs and Si@C NFs (**a**) before cycling and (**b**) after cycles (inset: equivalent circuit used to fit the experimental data).

Electrochemical impedance spectroscopy (EIS) measurements were carried out on the three samples, and the Nyquist plots before and after 20 cycles are shown in Figure 5. All the plots consist of one semicircle in the high-frequency range that is attributed to charge-transfer resistance and a sloping straight line in the low-frequency range is ascribed to the diffusion resistance of Li^+^. There is no doubt that the radius of the semicircle before cycling is slightly larger than that after the 20th cycle for Si@C NFs, which might be related to the permeation process between the fibers and the liquid electrolyte, indicating that a stable SEI layer can be formed. After cycling, the impedances of the Si/C NFs and the pure Si NP electrodes increase rapidly. The equivalent circuit (inset of Figure 5b) is used to further analyze the interfacial stability, where R_e_ and R_ct_ represent the ohmic resistance (the sum resistance of electrolyte, separator and electrical contacts) and the charge-transfer resistance, respectively. The double layer capacitance is labeled as CPE and the Warburg impedance Zw corresponds to the diffusion of lithium ions into the bulk electrodes. Table S1 shows the fitted resistance values of pristine Si NPs, Si/C NFs and Si@C NFs obtained from the equivalent circuit after cycling, the impedance of Si@C NFs (65.27 Ω) is much smaller than pure Si NPs (314.8 Ω) and Si/C NFs (115.85 Ω), indicating that core–shell structure effectively improve the stability of electrode while the bare electrode is unable to bear the volume expansion and accelerates the accumulation of the SEI film.

**Figure 6:**
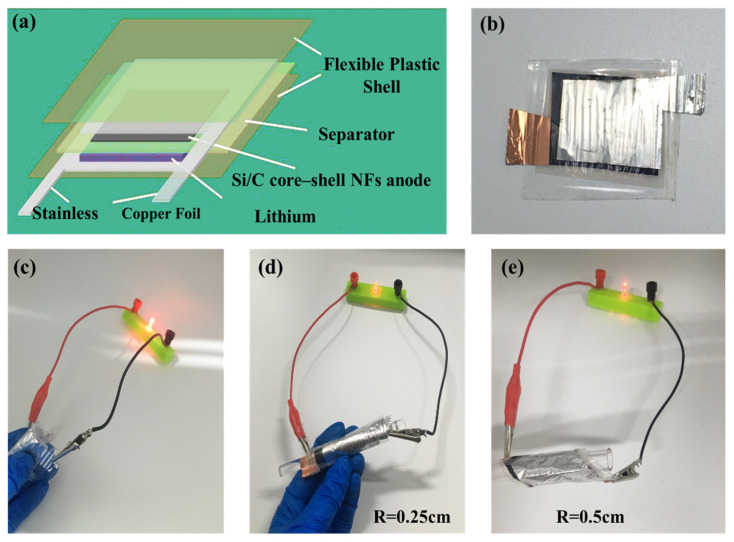
(**a**) Scheme of a flexible battery using Si/C core–shell mat as the anode and commercial lithium film as the counter electrode. Digital photographs of a flexible battery (**b**) and an LED lit by the flexible battery under bending (**c**) and enwinding (**d**,**e**) states.

The excellent electrochemical performance along with mechanical flexibility of Si@C NFs makes it promising for flexible electronics. We further demonstrate the fabrication of a flexible battery; a flexible LIB was designed with the Si@C NFs as the anode, lithium metal film as the counter electrode, and LiPF_6_ as the electrolyte (Figure 6a,b). The bent flexible battery exhibits a stable cycle performance without obvious capacity loss, as shown in Figure S3. Meanwhile, the as-fabricated flexible battery can easily light a commercial LED in a flat state or even under extreme deformation, such as bending at a radius of 0.5 and 0.25 cm, respectively, revealing that the electrical stability of the fabricated flexible battery is hardly affected by external bending stress (Figure 6c–e). Overall, the flexible battery possesses good capacity, cycle performance, and deformation ability by comparing with previous literature [30,31,32,33], the details are listed in Table S2.

## 4. Conclusions

In summary, we design a novel core–shell structured Si@C NFs anode with a hierarchical porous structure by embedding Si NPs in porous carbon core matrix and encapsuling by a hard carbon shell. As the core–shell structure can not only act as the buffer to mitigate the negative effects of volume expansion of Si NPs and avoid the direct explosion of Si NPs, but also provide efficient pathways to transport electrons and ions. This Si@C NFs anode exhibits a reversible capacity of 762.0 mAh g^−1^ after 100 cycles and a capacity of 479.7 mAh g^−1^ at a current density of 2 A g^−1^ is maintained. Additionally, the electrochemical performance of the flexible battery is hardly impacted under an extreme bending state, illustrating an excellent electrochemical performance along with the mechanical flexibility of Si@C NFs, making them promising for applications in flexible LIBs.

## Data Availability

Data that support the findings of this study are available from the corresponding authors upon reasonable request.

## Supplementary Materials

The following are available online at https://www.mdpi.com/2079-4991/11/12/3454/s1, Figure S1: The SEM images of (a) Si@C NFs and (b) Si/C NFs, Figure S2: The cyclic voltammograms curves (a) and galvanostatic charge/discharge profiles (b) of different cycles of the pure Si electrode, Figure S3: (a) CV curves at a scan rate of 0.1 mV/s and (b) charge/discharge curves at a current density of 0.1 A/g of flexible battery, Table S1: Values of equivalent resistance used for fitting the experimental date, Table S2: Parameters compare with previous literatures.
