# Supplementary material for: Coaxial Electrospinning Construction Si@C Core–Shell Nanofibers for Advanced Flexible Lithium-Ion Batteries
Source: Nanomaterials (Basel). 2021 Dec 20;11(12):3454. doi: 10.3390/nano11123454 (PMC8709299; doi:10.3390/nano11123454)
Supplement: Supplementary file 1 [file nanomaterials-11-03454-s001.zip › nanomaterials-1472439-supplementary.pdf]

nanomaterials

MDPI

Supporting Information

Coaxial Electrospinning Construction Si@C Core–Shell Nanofibers for Advanced Flexible Lithium-Ion Batteries

Li Zeng, Hongxue Xi, Xingang Liu * and Chuhong Zhang *

State Key Laboratory of Polymer Materials Engineering, Polymer Research Institute, Sichuan University, Chengdu 610065, China; zengli_0718@foxmail.com (L.Z.); hongxue@scu.edu.cn (H.X.)
* Correspondence: liuxingang@scu.edu.cn (X.L.); chuhong.zhang@scu.edu.cn (C.Z.)

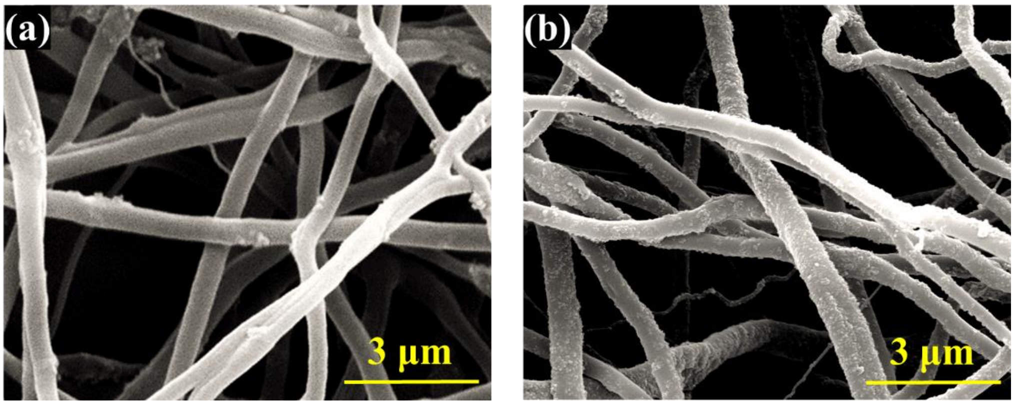

Figure S1. The SEM images of (**a**) Si@C NFs and (**b**) Si/C NFs.

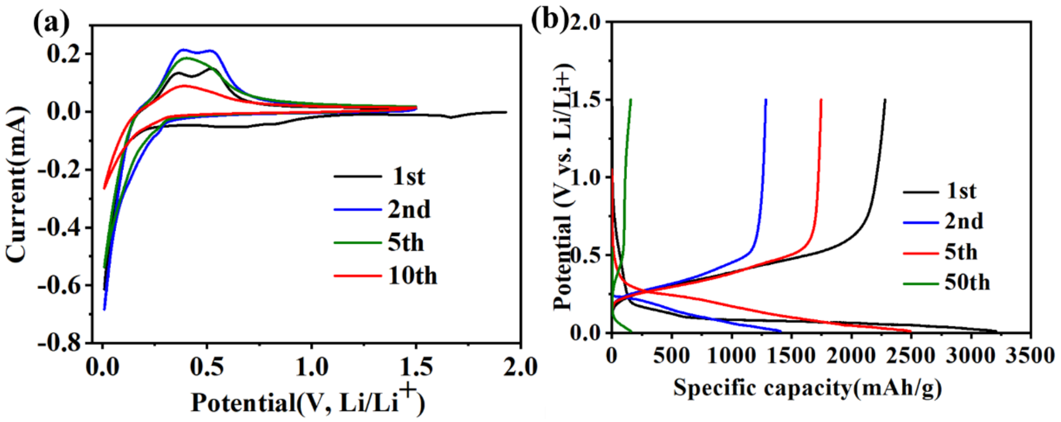

Figure S2. The cyclic voltammograms curves (**a**) and galvanostatic charge/discharge profiles (**b**) of different cycles of the pure Si electrode.

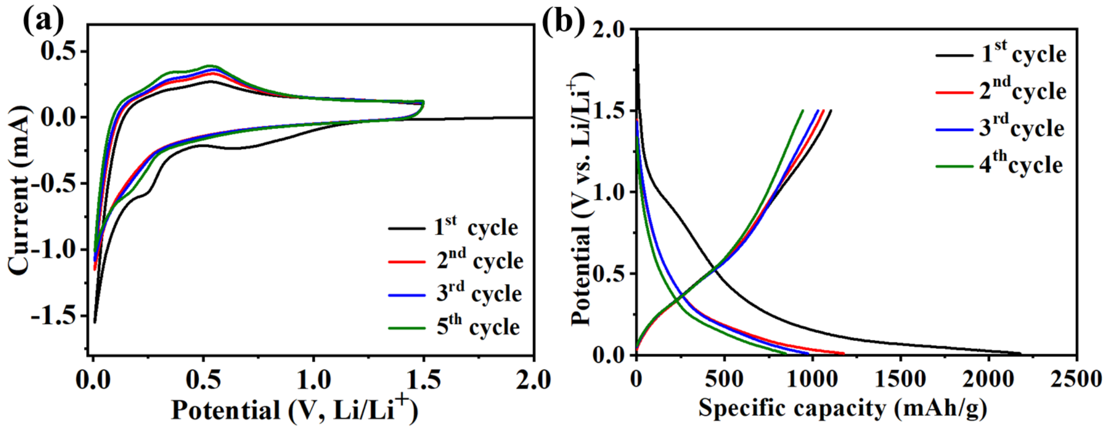

Figure S3. (**a**) CV curves at a scan rate of 0.1 mV/s and (**b**) charge/discharge curves at a current density of 0.1 A/g of flexible battery.

Table S1. Values of equivalent resistance used for fitting the experimental date.

Component	Fitted values		
	R_e/Ω	R_{ct}/Ω	R_{total}/Ω
**Pure Si NPs**	11.21	279.9	291.11
**Si/C NFs**	7.93	106	113.93
**Si@C NFs**	1.88	15.48	17.36

Table S2. Parameters compare with previous literatures.

	Capacity	Cycle Performance	Flexibility
**Ref.30**	936 mAh/g<br>@0.1 A/g	~80.5% after 100 cycle<br>@0.1A/g	No
**Ref.31**	1821 mAh/g<br>@0.2 C	~56.9% after1000 cycles<br>@2C	Yes
**Ref.32**	1381 mAh/g<br>@0.14 A/g	~100% after 100 cycle<br>@0.24A/g	No
**Ref.33**	1488 mAh/g<br>@0.2 A/g	~69.1% after 100 cycle<br>@0.5A/g	No
**This work**	1162.8 mAh/g<br>@0.2 A/g	~50.8% after 100 cycle<br>@0.1A/g	Yes
